# Heterogeneous Mixture of Amniotic Cells is Likely a Better Source of Stem Cells than Adipose Tissue

**DOI:** 10.1007/s00005-019-00538-5

**Published:** 2019-04-16

**Authors:** Diana Kitala, Agnieszka Klama-Baryła, Marcelina Misiuga, Wojciech Łabuś, Małgorzata Kraut, Michał Szapski, Marta Lesiak, Daniel Krakowian, Aleksander L. Sieroń, Marek J. Łos, Marek Kucharzewski

**Affiliations:** 1 Stanislaw Sakiel, MD Center for Burn Treatment, Jana Pawła II 2, 41-100 Siemianowice Śląskie, Poland; 2 Higher School of a Strategic Planning, Dabrowa Gornicza, Poland; 3 Silesian Medical School in Katowice, Katowice, Poland; 4 Department of General, Molecular Biology and Genetics, Medical University of Silesia, Katowice, Poland; 5 LinkoCare Life Sciences AB, Linköping, Sweden; 6 Biotechnology Centre, Silesian University of Technology, Chrobrego 8, 44-100 Gliwice, Poland; 7 Chair and Department of Descriptive and Topographic Anatomy, School of Medicine, Division of Dentistry in Zabrze, Medical University of Silesia, Zabrze Rokitnica, Poland

**Keywords:** Stem cells, Amniotic membrane cells, Adipose tissue cells, Burn treatment

## Abstract

Stem cells are increasingly being used in the course of burn treatment. As several different types of stem cells are available for the purposes, it is important to chose the most efficient and the most practicable stem cell type. The aim of this study was to compare the potential of heterogeneous amnion cell mixture with the presently used standard therapy, the adipose tissue-derived stem cells. The placenta was collected during a Cesarean section procedure. Adipose tissue tissue-derived cells were isolated using the Cytori’s Celution® System. Cells were tested for fulfillment of the minimum criteria for stem cells. The efficiency of cell cultures was tested by an analysis of population doubling, cell proliferation, cell cycle and cell migration. Amniotic cells presented a higher ability for differentiation to chondrocytes and osteocytes than adipose-derived regenerative cells but a lower ability for differentiation toward adipocytes. Additionally, in vitro experiments have demonstrated a higher applicability of amniotic cells than adipose tissue-derived stem cells. Amniotic cells show several advantages: easy access to placenta, low costs and a lack of ethical dilemmas related to stem cell harvesting. The main disadvantage is, however, their availability, as isogenic treatment would only be possible for women around children-bearing age, unless personalized banks for amniotic cells would be established.

## Introduction

The aim of burn wound treatment is the structural and functional restoration of damaged skin to its original state (Lataillade et al. 2007; Sorg et al. 2012). The use of stem cells in burns treatment improves wound healing, understood as an earlier wound closure, healing acceleration, prevention of scar contractures, skin regeneration and, at best, its appendages (Blais et al. 2013; Nelson et al. 2009).

The stem cells, when applied in the treatment of burns, reduce systemic inflammatory response and, thus, reduce the risk of infectious complications and improve the treatment outcome of patients (Branski et al. 2009; Teng et al. 2014). Nowadays, several types of stem cells and partly differentiated tissue components are available (Cieslar-Pobuda et al. 2017), as well as novel artificial extracellular matrix components (Hudecki et al. 2017). However, an optimal source of stem cells and methods of applications in burn patients has to be specified (Blais et al. 2013). Previous research showed that mesenchymal stem cells (MSCs) have a tremendous regenerative potential. The benefits of the use of the stem cells are partly related to their paracrine activity (Teng et al. 2014). Unlike medication, acting on particular pathways, MSC shows therapeutic activity by acting upon the combination of mutually linked pathways (Lataillade et al. 2007; Potten and Loeffler 1990). It has been proven that the presence of bone marrow stem cells restores angiogenesis by increasing basic fibroblast growth factor and vascular endothelial growth factor levels and by an intensification of collagen synthesis, which is significantly higher in bone marrow stromal cells than in skin fibroblasts. Moreover, using allogeneic, fibroblast-like MSC on burn wounds under skin grafts reduces post-surgery pain and also promotes angiogenesis and epithelial growth (Teng et al. 2014). Despite the fact that an intensified expression of connective tissue growth factor in MSCs is limited to early phases of tissue repair, it improves the overall process (Steigman et al. 2008). The immunosuppressive character of MSC has a vital role in the case of allogeneic grafts, because the “immunologically privileged” cell population can be used to reduce the frequency of occurrence and the intensification of the graft-versus-host disease (GVHD) (Machado Cde et al. 2013). There is clinical evidence that intravenous infusions (2 × 10^5^ cells/kg) of allogeneic MSCs reduce the GVHD (Machado Cde et al. 2013). Second-degree burn is accompanied by the destruction of all epidermal layers. The first clinical trial of using MSCs in burn treatment was performed in Russia in 2005. A female patient with burns affecting 40% total body surface area (incl. 30% being third-degree burns) was treated with allergenic bone marrow mesenchymal stromal cells which led to increased angiogenesis and an accelerated wound healing process. The therapeutic application of MSC gives a positive outcome also in post-radiation burn treatment. In 2007, autologous MSCs were applied to a patient, resulting in the reduction of inflammation response and an improved healing process of the burns (Lataillade et al. 2007). An application of regenerative cells derived from adipose tissue becomes increasingly popular in regenerative medicine. Those cells can be isolated by liposuction and such process allows for collection of clinically significant quantities of such cells without ethical concerns (Arana et al. 2013; Kuhbier et al. 2010; Strioga et al. 2012). Multiple cycles of lipoaspirate rinsing and enzymatic digestion result in obtaining a stromal vascular fraction—a heterogeneous mixture containing, i.e., MSC, endothelial cells and fibroblasts (Arana et al. 2013; Kuhbier et al. 2010). Unfortunately, the most significant limitation of this method is the fact that 300 g is the minimal volume of patient-derived adiposal tissue necessary for performing the procedure. A single procedure is limited to the group of overweight patients. Moreover, the number of stem cells depends on the volume of collected tissue and the procedure of cell expansion under in vitro conditions is typically not being performed. The above-mentioned limitations prompted us to search for suitable stem cells sources. Placenta is a convenient source of large qualities of stem cells (Kim et al. 2012; Paracchini et al. 2012; Teng et al. 2014). So far, amnion was used for wound healing in a mouse model (Steigman et al. 2008).

The aim of this study was to compare the potential of a heterogeneous amnion cell mixture with presently used golden standard therapy—adipose-derived regenerative cells (ADRCs; a heterogeneous mixture of adipose tissue cells) obtained using commercially available devices. In this study, we checked a number of cells, which can be obtained from a 21-day culture. This is an average time necessary to perform skin cell grafting in burn patients. Due to positive results of serological tests, some patients, much to their dismal, are not accepted for establishing cell cultures of autologous keratinocytes. Other stem cell sources offer an alternative for such patients. Data analysis was targeted on verification of a clinical potential of mixtures of cells isolated from two sources in burn treatment.

## Materials and Methods

### Placental Cell Isolation

The proposed research program received a positive opinion of the Bioethics Committee of the Silesian Provincial Medical Chamber.

The placenta was collected during a Cesarean section procedure under operating theatre conditions. Before the collection of the placenta was performed, the patient’s informed consent was obtained in accordance with the protocol accepted by the bioethical commission.

The donor was tested for: human immunodeficiency virus (HIV-1 and HIV-2), surface antigen of the hepatitis B virus, hepatitis C virus and syphilis; all tests turned negative. The placenta preparation was carried out in a tissue bank clean room (GMP class C; ISO class 7) under laminar low cabinet conditions (GMP class A; ISO class 4.8; HSKS 18, thermo). The material remaining after the placenta preparation procedure was placed in a sterile container (Maco Biotech Freezing Storage Pots 40–80 ml, Macopharma, France) and moved to an in vitro cell culture clean room with class B air purity, where the primary cell culture has been carried out.

All procedures linked with cell material processing were performed in a laminar flow cabinet (class A). Cells were isolated by mechanical homogenization of amniotic membrane. Obtained homogenizate was squeezed through a sieve with 70 µm pores (BD Falcon, BD Biosciences, USA). Cellular suspension was quantified (Tali® Image-Based Cytometer, Thermo Fisher Scientific, USA) and then 100,000 cells were seeded into a cell culture bottle of 75 cm^2^ (Sarstedt, Germany). The bottle was then filled with 15 ml of medium StemXVivo Serum-Free Human MSC Expansion Media (R&D Systems, USA). For research, waster material from five placentas was used. Adiposal tissue-isolated cells were derived using Cytori’s Celution® System (Cytori Therapeutics, Inc., USA). Five patients underwent an autologous cell application procedure.

The isolated cells were rinsed with a normal phosphate-buffered saline solution (PBS; CytoGen—Polska Sp. z o.o., Poland) and counted (by a Tali® Image-Based Cytometer). The cell culture was carried out at 37 °C temperature, 95% humidity and 5% CO_2_ to obtain 80% confluence and then detached [using TrypLE™ Select (IX) solution by Phenol Red Life Technologies, Thermo Fisher Scientific, USA]. Such prepared cells were then used for experimental procedures.

### Fulfilling the Minimum Criteria for Stem Cells

Cells were evaluated after a column-based method of isolation (primary passage) and before grafting, using the following tests.

#### Adherence to Plastic and Expression of Selected Lineage Markers

Evaluation of adherence to plastic was performed by a microscopic observation using a motorized inverted microscope Olympus IX81. Cells were stained with α1 integrin conjugated with Alexa Fluor 350 dye (Thermo Fisher Scientific, USA). Cells were stabilized with 3.7% formaldehyde solution in PBS for 30 min and then permeabilized by 20-min incubation with 0.5% Triton X-100 solution in PBS. Three percent bovine serum albumin solution in PBS was a wash buffer. We used a 1 µl of antibody for each well (cell culture well plate). The time of incubation with the antibodies in total darkness was 45 min. The intensity of a histochemical reaction was estimated with “Score”, a semi-quantitative method (Litwin and Gajda 2011), in accordance with the intensity criteria listed below: 0—invisible stain, 1—visible staining, 2—weak, but noticeable staining, 3—moderately intense staining, 4—very intense staining. Hundred cells randomly chosen from each preparation were assessed. The results were calculated based on the following equation: staining intensity = staining grade (*d*) × number of cells (*n*). Microscopic observation was performed using a motorized inverted microscope Olympus IX81, and photos were taken using the Cell M program (Olympus, Japan).

#### Differentiation into Cell Lineages Typical for All Three Germ Layers

The analysis was performed using the Human Mesenchymal Stem Cell Functional Identification kit (R&D Systems, USA). Supplements and growth factors enable a differentiation of human MSCs into adipocytes, chondrocytes and osteocytes. An antibody validation panel contained the goat anti-mouse antibody FABP4, the goat anti-human agrecane, and the mouse anti-human osteocalcine. Cells were seeded and cultured in accordance with the Adipogenese Protocol: cells seeded in volume 3.7 × 10^5^ and cultured using αMEM medium with adipocyte differentiation supplements. The medium was changed every 72 h. After 14 days, culture cells were fixed with 3.7% formaldehyde solution in PBS and staining was performed in accordance with the procedure described for the α1 integrin.

#### An Analysis of Cellular Markers Typical for MSCs

A surface marker analysis was performed using the flow cytometry technique. Cells were analyzed by the BD Stemflow™ hMSC Analysis Kit (BD Biosciences, USA) in accordance with the manufacturer’s protocol. According to the protocol, multipotent stem cells are positive for CD105, CD73 and CD90 markers at a minimum level of 95% with a negative expression of hematopoietic markers (< 2% of positive cells).

### The Assessment of the Quality of Cultured Cells

Sample of isolated cells were preliminarily tested. The evaluation of the optimal method of stem cells isolation was based on the results of quantitative tests of the obtained cells, the results of vitality evaluation and the percentage of apoptotic cells in cultures. An analysis was performed in accordance with the manufacturer’s protocol, using the Tali® Dead Cell Red set on Tali® Image-Based Cytometer (Life Technologies, Thermo Fisher Scientific, USA). Moreover, we performed the analysis of a long-term impact on an isolation method, by assessing after 3 weeks, the kinetics of growth and cell population doubling.

#### An Analysis of the Population Doubling

To analyze the multiplicity of population doubling (PD), the cells were detached from the surface of cell culture bottle by digestion [using TrypLE Select Enzyme (1×) by Life Technologies] after the scheduled end of the culture’s growth (21 days) and compared with the number of seeded cells. The doubling time of population was calculated using the formula:

$$PD\;=\;\frac{{\left( {\log {N_h} - \log {N_0}} \right)}}{{\log \left( 2 \right)}}$$

where ${N_h}$ is the number of cells on the day of the end of the growth of the cell culture and ${N_0}$ is the cell-seeding number.

#### An Analysis of Cell Proliferation

The set of The Click-iT® EdU Alexa Fluor® 488 Imaging Kit uses the nucleoside analogue of thymidyne. The test was performed in accordance with the manufacturer’s recommendations. Hundred-thousand cells were seeded in a six-well plate to compare the proliferation abilities of amnion cells, regenerative cells and adipose-derived stem cells (ADSC). Stabilization and staining were carried out on the seventh day after the seeding.

#### An Analysis of Cell Cycle

The analysis was performed using The Tali® Cell Cycle Kit. Cells were seeded in a six-well plate at a density of 500 000 cells/well. The experiment was undertaken in accordance with the manufacturer’s protocol. The cells were detached from the plate [TrypLE™ Select (1×), Phenol Red Life Technologies] at the seventh day of the culture, and analyzed.

### Assessment of Cell Migration Speed: Wound-Healing Assay

The wound-healing assay was performed using the CytoSelect kit. The experiment was conducted in accordance with the manufacturer’s protocol. Five-hundred-thousand cells/well were seeded. Cell migration was observed at 30-min intervals. Total coverage of a test-generated wound was considered as an end of the migration process.

### Statistical Analysis

Statistical analysis was performed using the STATISTICA 10 software. The assumptions of normal distribution were analyzed with the Shapiro–Wilk test. The assumptions of the equality of variance were checked with the Levene’s test. Statistical hypothesis testing for two independent samples was performed using the Mann–Whitney *U* test. The Kruskal–Wallis test was used for performing a comparison of more than two groups of independent samples, which did not meet the normality assumption. The parametric equivalent of the Kruskal–Wallis test was a one-way analysis of variance (ANOVA). For an equal variance test, a post hoc Tukey’s test was performed, and for different variances, the Games–Howell’s test. The significance level was set at 0.05 (5%).

## Results

### Fulfilling the Minimum Criteria for Stem Cells

**Fig. 1:**
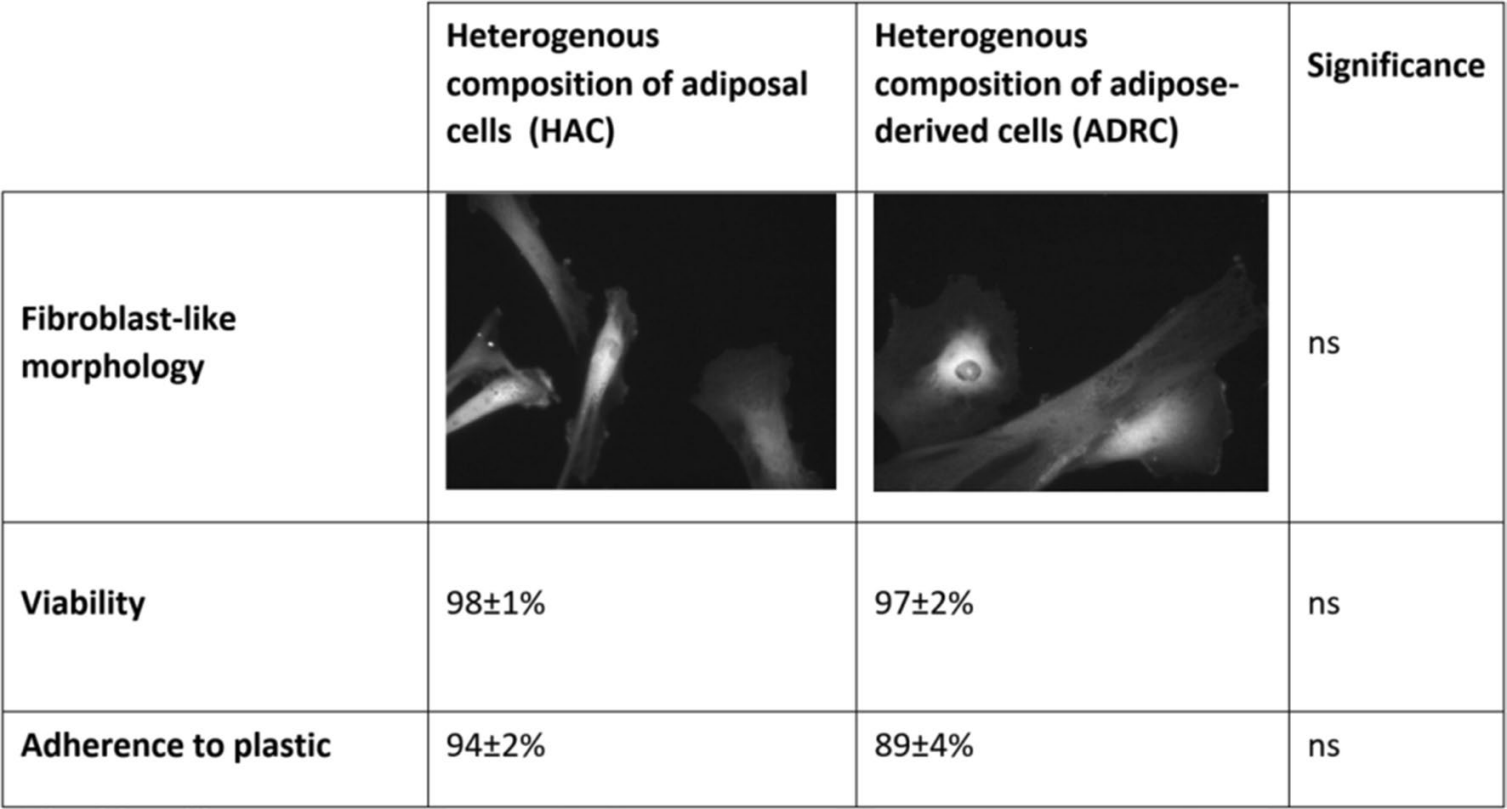
Comparison of fulfilling of the minimum criteria for the multipotent stem cells in adipose- and amnion membrane-derived isolates

Based on the analyses, we concluded that both the heterogeneous mixture of amniotic cells and the ADRCs demonstrated fibroblast-like morphology (Fig. 1).

There were no significant differences in cell viability analysis (*p* ≥ 0.05) and their abilities to adhere to plastic (cell culture bottle; *p* ≥ 0.05). Moreover, there were no significant differences in the ability for differentiation into all cells characteristic for three germ layers (Table 1).

**Table 1:** Differentiation potential into cell typical for all three germ layers

Differentiation into three mesodermal cell lineages	Osteogenesis	41 ± 6	40 ± 4	ns
	Adipogenesis	89 ± 13	111 ± 6	ns
	Chondrogenesis	157 ± 14	143 ± 9	ns

*ns* not significant

Amniotic cells presented a higher ability for differentiation than chondrocytes and osteocytes. However, they differentiated towards adipocytes at lower rate than ADRC.

The analysis of multipotent cell markers showed no significant differences in the quantity of the CD90 marker expression (*p* ≥ 0.05) between ADRCs and a heterogeneous mixture of amniotic cells. In the case of CD105, statistically significant differences between the analyzed cell types (*p* = 0.043) were reported (Table 2).The lowest value was observed for ADRCs (49.5%). However, no statistically significant differences in the ability to differentiate were observed in the population of cells expressing the CD73 marker (*p* ≥ 0.05). We observed no substantial differences in the population of cells showing the presence of hematopoietic stem cell markers in tested cells (*p* ≥ 0.05). Moreover, the CD44-experssing cells, derived from all sources tested, show similar differentiation potential (*p* ≥ 0.05).

**Table 2:** Presence of markers typical for mesenchymal cells

Presence of cell markers for mesenchymal cells	CD90: 97.1 ± 2%	CD90: 94.4 ± 2%	*p* < 0.05
	CD105: 74.8 ± 1%	CD105: 49.5 ± 1%	
	CD73: 99.1 ± 0.5%	CD73: 96.7 ± 1%	
	HSC: 1.2 ± 0%	HSC: 7.11 ± 0%	
	CD44: 97.8 ± 2%	CD44: 98.3 ± 2%	

*HSC* hematopoietic stem cell

The results of the analysis performed after the first passage suggests that both the heterogeneous mix of amniotic cells and the adipose-derived cells show abilities for differentiation into adipocytes, chondrocytes and osteocytes after 21 days.

### Assessment of Cell Proliferation and Migration

**Fig. 2:**
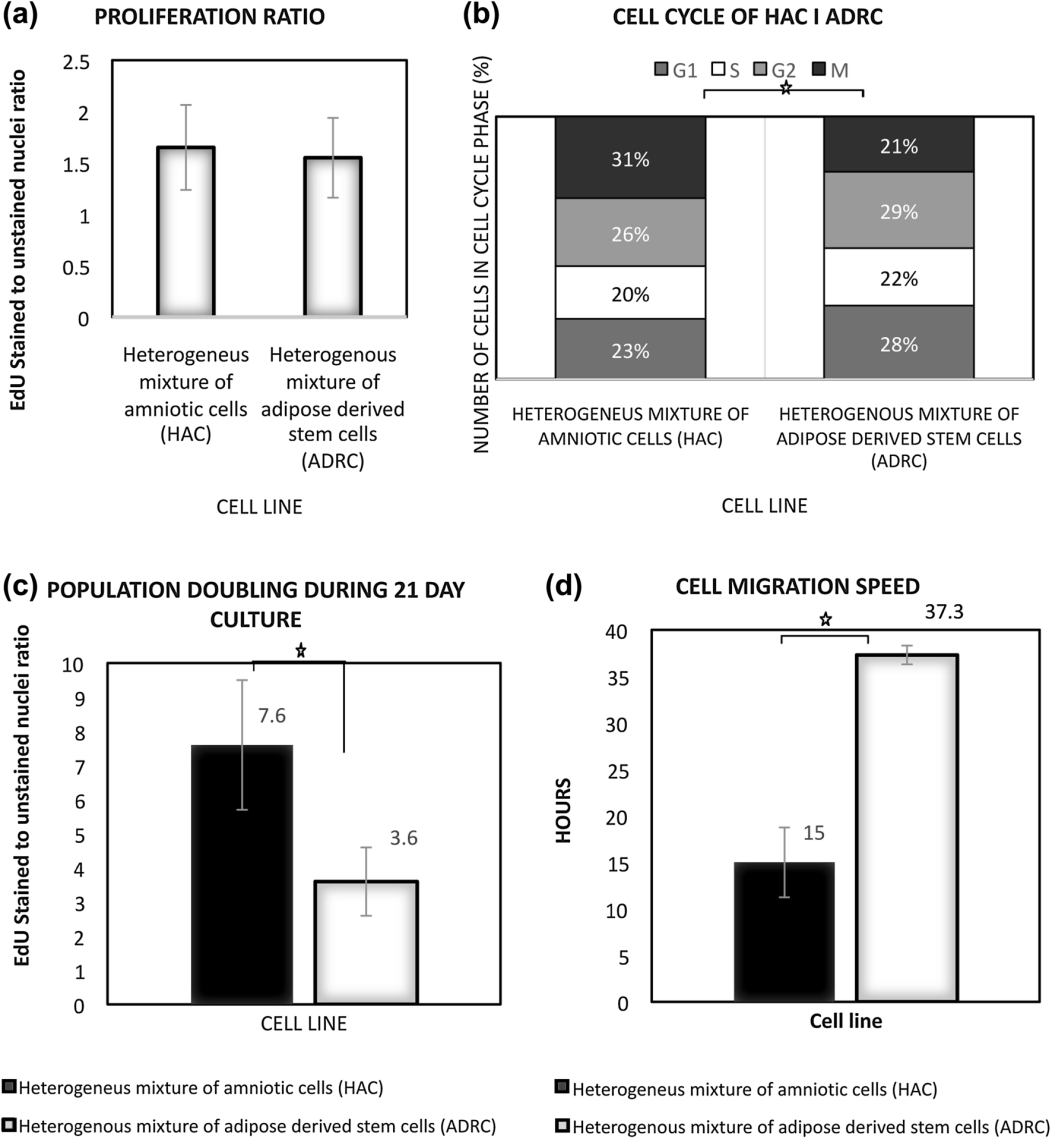
**a** No differences in approximate number of proliferative cells of both cultures were reported on the 7th day. **b** Significantly more cells were observed in the mitosis phase of the amniotic cell culture. **c** The number of population doubling in the amniotic cell culture in the 21st day after the seeding has significantly increased. **d** The average ± SD speed of amniotic cells migration was higher than that of the adipose-derived regenerative cells (ADRCs)

The heterogeneous mixture of amniotic cells exhibited shorter G1 phase as compared to the ADRC (approx. 23%; Fig. 2; *p* = 0.002). We have observed no differences in number of cells in phases S and G2 (*p* ≥ 0.05). However, the proportion of cells undergoing mitosis was significantly (*p* = 0.002) higher in heterogeneous mixture of amniotic cells as compared to ADRC where 21% of population was in mitosis. Cell proliferation assay revealed no differences in proliferation capacity of both cell populations; the number of proliferating cells compared to non-proliferating cells [EdU(+)EdU(–)] was similar. PD time was used for an evaluation of a long-term proliferative capacity. This analysis showed that a significantly higher number of doubling occur in human amniotic cell cultures (*p* < 0.001), as compared to ADRC cultures. Cells from a heterogeneous mix of amniotic cells had the capacity to close the “wound” faster than ADRC (*p* = 0.0).

## Discussion

Tissue engineering as a fast-growing branch of science offers new perspectives on therapeutic approaches towards wound healing (Chen et al. 2009; Kucharzewski et al. 2019). Especially in the case of extensive burns, such as, i.e., III-burns, human stem cells seem to be increasingly considered as a way of therapy (Mansilla et al. 2015; Rasulov et al. 2005). Similarly, a stem cell therapy for chronic wounds is even more frequently propounded (Lee et al. 2016). Adipose tissue is a popular source of stem cells; however, the number of stem cells depends on the adipose tissue volume collected, and the adipose tissue availability for collection depends on the severity of the burned patient’s health status. The above-mentioned limitations implicate the need of other tissues as sources of stem cells. Hence, the amniotic stem cells, containing two types of cells, human amniotic epithelial cells and human mesenchymal stem cells, are gaining interest as the potential sources of stem cells for clinical applications. Both types of cells originate from the pregastrulation stage of an embryo’s development before the separation of three embryonal germ layers and their majority express epithelial features (Machado Cde et al. 2013). Amniotic cells could remain undifferentiated although they are able to differentiate into all three germ layers (Pirjali et al. 2013). Additionally, they are less prone to form teratomas after implantation into immunodeficient mice. This particular feature, differentiates the amniotic stem cells from embryonic stem cells and indicates similarities with adult stem cells. However, a cytometric analysis of a heterogeneous culture showed that only 74.8% of amniotic cells expressed the CD105 cell marker that was probably caused by an admixture of amniotic epithelial cells in culture. It should be noted, however, that in an ADRC culture, the number of cells presenting an expression of that marker was significantly lower than 49.5%. It means that amniotic cell culture is much more homogeneous than the culture of regenerative- and stem cells (ADRC) separated using a Cytori’s Celution® System commercial kit. Both cultures do not fulfill minimal stem cell criteria released by the International Society for Cellular Therapy in 2006 despite their adherence to plastic and an ability to differentiate into cells of lineages typical for all three germ layers (Potten and Loeffler 1990). Nevertheless, adipose cells do express CD90 and hematopoietic markers at an adequate level.

We have discovered that compared to ADSCs, a very high expression of insulin-like growth factor 1 in amniotic cells is particularly important as it likely affects the speed of wound closure. Moreover, amniotic cells promote the migration of other cells, which is of crucial importance in wound healing (Kim et al. 2012). These results were confirmed by the test mimicking the wound healing process under in vivo conditions, namely the CytoSelect™ 24-Well Wound-Healing Assay. It has thus been proven that amniotic cells have the ability to close wounds in vitro 2.5 times faster than adipose-derived cells. Human amniotic stem cells demonstrate an accelerated proliferation compared to MSC collected from other sources (bone marrow, adipose tissue) (Mizokami et al. 2009; Veryasov et al. 2014). This conclusion is confirmed in this study; however, there is no statistically significant difference in the short-term test (7 days). At the same time, however, the difference between amniotic cells and ADRC in the number of population doublings in a 21-day culture was reported. The analysis of cell cycle phases (the number of cells in a mitotic phase) can confirm that ADRCs present a significantly higher proliferation ability than amniotic cell.

In conclusion, our in vitro data demonstrated a higher applicability of amniotic cells than adipose-derived cells for clinical applications involving wound healing. Amniotic cells have additional, price-based advantage—the cost of amnion collection (a medical waste material) is low. Easy access to placenta, the lack of ethical constraints and the inability to form teratomas in vivo make them a plausible, promising alternative for tissue engineering applications (Kong et al. 2013; Lee et al. 2016; Placzek et al. 2009; Rennie et al. 2012). This study shows the purposefulness of applying this source of cells, particularly in patients in whom, due to their overall health condition, or to low body mass, liposuction procedures cannot be performed.

It should be noted, however, that the development of a network of Tissue Banks that collect amniotic cells is required. Such amniotic cells should be considered as an advanced therapy medicinal products that may supplement (provide additional options for) clinicians willing to apply stem cells also from adipose tissue, separated with the Cytori’s Celution® System. Adipose tissue-derived cells are a heterogeneous population with a low content of stem cells, and lower abilities for wound healing. The single-dose cost is, however, higher than in amniotic cell therapy; authors thus suggest using the Cytori’s Celution® System only in case when the medical care facility does not have access to a Tissue Bank, or the clinical situation requires performing a single surgical procedure.

## Abbreviations

ADRC: Adipose-derived regenerative cell
ADSC: Adipose-derived stem cell
PD: Population doubling
GVHD: Graft-versus-host disease
MSC: Mesenchymal stem cell
PBS: Phosphate-buffered saline
